# MV-MFF: Multi-View Multi-Feature Fusion Model for Pneumonia Classification

**DOI:** 10.3390/diagnostics14141566

**Published:** 2024-07-19

**Authors:** Najla Alsulami, Hassan Althobaiti, Tarik Alafif

**Affiliations:** Department of Computer Science in Jamoum, Umm Al-Qura University, Makkah 25371, Saudi Arabia; hmthobaiti@uqu.edu.sa (H.A.); tkafif@uqu.edu.sa (T.A.)

**Keywords:** pneumonia, multi-view, variational autoencoder, chest X-ray, CheXpert, image classification

## Abstract

Pneumonia ranks among the most prevalent lung diseases and poses a significant concern since it is one of the diseases that may lead to death around the world. Diagnosing pneumonia necessitates a chest X-ray and substantial expertise to ensure accurate assessments. Despite the critical role of lateral X-rays in providing additional diagnostic information alongside frontal X-rays, they have not been widely used. Obtaining X-rays from multiple perspectives is crucial, significantly improving the precision of disease diagnosis. In this paper, we propose a multi-view multi-feature fusion model (MV-MFF) that integrates latent representations from a variational autoencoder and a β-variational autoencoder. Our model aims to classify pneumonia presence using multi-view X-rays. Experimental results demonstrate that the MV-MFF model achieves an accuracy of 80.4% and an area under the curve of 0.775, outperforming current state-of-the-art methods. These findings underscore the efficacy of our approach in improving pneumonia diagnosis through multi-view X-ray analysis.

## 1. Introduction

Pneumonia, a severe lung infection, poses significant risks to respiratory health. A swelling from pus and fluid brought on by pneumonia makes breathing difficult and reduces oxygen absorption in human bodies. According to a report published by the World Health Organization, pneumonia in recent years has caused the death of 14% of children under the age of five [1]. Chest X-rays have emerged as a key tool for diagnosing pneumonia and various lung, heart, and other disorders [2]. However, accurate interpretation relies heavily on the expertise of radiologists, who can distinguish pneumonia from other conditions. Nevertheless, there might still be challenges in distinguishing pneumonia from similar lung diseases.

Numerous research endeavors have aimed to leverage artificial intelligence models for pneumonia diagnosis, capitalizing on advancements in medical image analysis. These efforts have led to the development of several models adept at accurately detecting pneumonia. Additionally, preprocessing techniques such as resizing images and data augmentation have played a pivotal role in enhancing detection accuracy. Previous studies have focused on diagnosing heart and lung conditions, including pneumonia, primarily using frontal-view X-rays. Frontal-view X-rays lack detailed information crucial for accurate diagnosis, primarily due to obstructions from heart structures, the diaphragm, and blood vessels, which obscure parts of the lungs in the frontal view. Contrarily, lateral-view X-rays offer a comprehensive depiction of the lung’s total volume, making them invaluable for detecting lesions not visible in frontal views. Consequently, incorporating both frontal and lateral X-ray images is essential for a dependable diagnosis [3]. Currently, radiologists depend on multi-view radiographs to gather detailed disease information and ensure accurate classification.

Variational autoencoders (VAEs) have demonstrated effectiveness in unsupervised learning endeavors [4]. Numerous studies have leveraged the latent representation derived from VAEs to fulfill their objectives. The VAE aims to generate a rich latent representation capable of capturing useful features essential for subsequent image analysis tasks such as classification, segmentation, and prediction. Incorporating the β parameter into a VAE serves to adjust the balance between reconstruction and the disentanglement of latent representations [5,6].

In this research, we introduce a multi-view multi-feature fusion (MV-MFF) model, which integrates a VAE and a β-VAE to derive a latent representation crucial for pneumonia classification from multiple viewpoints. Additionally, we opted for the pretrained DenseNet121 CNN architecture to extract features for input into the β-VAE encoder. This decision stemmed from DenseNet121’s efficacy in extracting significant features in prior studies [7,8,9,10]. Our model’s objective is to leverage the integration of diverse features extracted from two distinct models to enhance performance in pneumonia detection.

The following is a summary of our work’s primary contributions:

- The reliability of pneumonia diagnosis is enhanced by the MV-MFF model, which takes advantage of latent features extracted from frontal and lateral X-rays in order to classify the presence or absence of pneumonia.
- Disentanglement methods are explored by adjusting the β value and evaluating its impact on classifying the presence or absence of pneumonia using multi-view images from the public CheXpert dataset.

This paper’s remaining sections are arranged as follows. In Section 2, the relevant literature is provided, and in Section 3, the public CheXpert dataset is reviewed. In Section 4, DenseNet121, variational autoencoder, and β-variational autoencoder models are reviewed. In Section 5, our approach is presented. The experimental setting, the results, and a comparison are presented in Section 6. Ablation study findings are in Section 7, discussion is in Section 8, and our work is concluded in Section 9.

## 2. Related Work

Many studies used frontal-view chest X-rays to classify pneumonia. Janizek et al. [11] applied an adversarial approach to solve the generalization problem for data outside the training set. They trained the model on the CheXpert dataset from the anterior–posterior (AP) and posterior–anterior (PA) views, excluding all the lateral views. The MIMIC-CXR dataset was also used for testing. An adversarial training method was proposed to enhance pneumonia detection from chest radiographs, utilizing two networks: a classifier for pneumonia presence prediction and an adversary for radiograph appearance prediction based on the classifier’s output. The model, according to the AUC, achieved a result of 0.747 on the test data from the CheXpert dataset, and using the MIMIC-CXR as external data, it achieved a result of 0.739. Recently, there has been some progress in the field of analyzing X-ray images from multiple views. Zhu et al. [10] presented a multi-view chest radiograph classification network (MVC-Net), which consisted of three branches that fused the frontal and lateral image views. Two branches extracted 2D feature maps in both frontal and lateral images, and a back-projection transfer (BPT) branch converted 2D feature maps to 3D. They used the MIMIC-CXR dataset, and the proposed approach achieved 0.693 for the frontal view, 0.669 for the lateral view, and 0.715 for multi-view using the AUROC metric.

Rubin et al. [9] suggested a new DualNet architecture for the classification of chest diseases by multi-view X-rays. Two DualNet networks were trained: one with PA–lateral pairs and the other with AP–lateral pairs. The underlying architecture was based on a DenseNet121 core that replaced the three-channel (RGB) input layer with a single-channel grayscale enhancement layer. To achieve the purpose of the proposed approach, they trained the model on the MIMIC-CXR dataset. To evaluate the model, the AUC criterion was used, achieving a score of 0.625 on DualNet PA–lateral and a score of 0.593 on DualNet AP–lateral for the classification of pneumonia. Likewise, Ma et al. [12] proposed cross-attention networks (CAN) to solve the data imbalance problem. After preprocessing, the images were pumped through two grids to extract the features. The image passed through convolutional and ReLU layers until the features were extracted. The Hadamard product was used to obtain multi-attention feature maps focusing on the area of infection. The proposed approach was tested on the frontal and lateral views of the CheXpert dataset. According to the AUC criterion, it achieved a score of 0.666 for the pneumonia classification task. Peng et al. [8] proposed a model based on two-stage feature extraction and feature fusion. In the first stage, they used DenseNet121, Inception V3, and Xception models to extract features from anteroposterior (AP), posteroanterior (PA), and lateral (LA) chest X-ray images. Multi-view features were combined, and the fully connected and softmax layers were added to obtain a pneumonia classification. The model achieved an AUC score of 0.71 on the MIMIC-CXR dataset.

Tulder et al. [13] introduced a cross-view transformer method for analyzing multi-view images. Their method used a view transformer to connect multiple views at the level of spatial feature maps instead of combining them after global pooling. To extract features from each view, they used ResNet-18 and then a cross-view transformer to transfer features between views. Their model was trained on the CBIS-DDSM and CheXpert datasets. They compared the single-view models and the late-join approach with the proposed cross-view transformers. Using the AUC scale, the frontal view achieved 0.75, the lateral view achieved 0.74, the late-join achieved 0.76, and the cross-view achieved 0.75 for the classification of pneumonia. The proposed approach achieved good results in some lung and heart diseases, but in pneumonia, late-join was better.

Multi-view X-rays have been widely employed in various medical applications beyond pneumonia classification. For instance, Kim [14] proposed the CheXFusion model for classifying medical images, considering the co-occurrence of diagnostic findings and multiple views available for each patient. Initially, the model trains a convolutional neural network (ConvNeXt) as a backbone to extract features from multiple views. These extracted features are then used as input to a transformer-based fusion module. This module leverages attention and self-attention mechanisms to effectively aggregate multi-view data for multi-label classification. The images are resized to 1024 × 1024, and the AdamW optimizer is employed. The model achieved an AUC of 0.850 on the MIMIC-CXR test set. Yuan et al. [15] proposed a generative decoder model that leverages multi-view chest X-rays to generate reports. They pretrained a Resnet-152 encoder on a large dataset to recognize 14 radiographic observations. Using a delayed fusion method with sentence-level attention, they synthesized multi-view visual features. To enhance the LSTM decoder’s descriptive capability and accuracy in medical contexts, they extracted common medical concepts from radiology reports and integrated them into the decoding process with word-level attention. Using the CheXpert dataset, they achieved a 0.764 AUC with cross-view consistency and multi-view fusion. Yang et al. [16] introduced an innovative bidirectional image Mamba (BI-Mamba) model designed to efficiently process multi-view high-resolution chest radiographs for predicting cardiovascular disease (CVD) risk. The BI-Mamba model enhances the traditional unidirectional Mamba by incorporating reverse directional information. This is achieved through parallel forward and backward blocks that operate in a recurrent manner. This architecture enables the model to capture extensive long-range information, which is then condensed into a classification token for CVD risk prediction. Utilizing the National Lung Screening Trail (NLST) dataset, the BI-Mamba model achieved an AUC of 0.8243.

Despite the existence of research that classifies pneumonia from multiple perspectives, as shown in Table 1, the methods used are still unable to achieve satisfactory results. This may be attributed to their reliance on simplistic models that extract specific features, ultimately leading to suboptimal classification performance. Our proposed approach is based on the use of variational autoencoder models to capitalize on latent representations, facilitating the integration of extracted features from the latent space for classification. This enables the classifier to gain a deeper understanding, leading to more effective classification.

## 3. Materials and Methods

### 3.1. CheXpert Dataset

**Figure 1:**
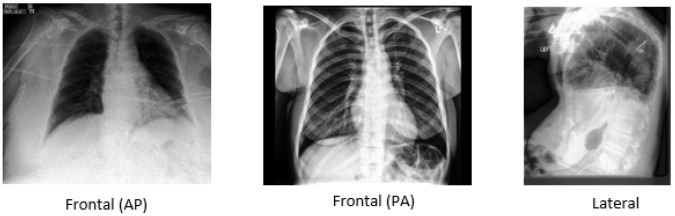
(**Left**): anteroposterior (AP) frontal view. (**Middle**): posteroanterior (PA) frontal view. (**Right**): lateral view (LA).

CheXpert [17] includes two frontal views, posteroanterior (PA), anteroposterior (AP), and one lateral (LA) view of chest X-ray images, as shown in Figure 1. Stanford Hospital provided the dataset between October 2002 and July 2017. The dataset covers 224,316 images gathered from 65,240 patients. The dataset comprises numerous observations related to lung and heart conditions, including atelectasis, enlarged cardiomegaly, fracture, lung opacity, lung lesion, pneumonia, pneumothorax, etc. Labels were categorized as positive, negative, uncertain, or missing values.

### 3.2. Preliminaries

#### 3.2.1. DenseNet121

**Figure 2:**
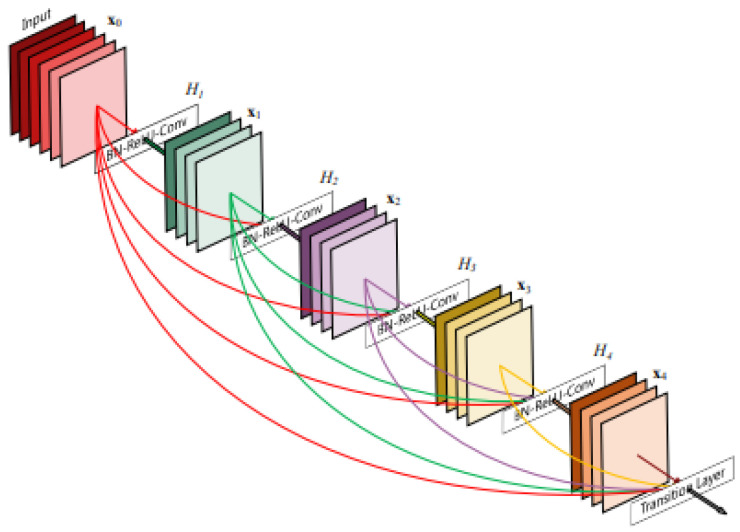
DenseNet architecture.

Convolutional neural networks (CNNs) are being used increasingly, and as a result, they are becoming deeper and more complicated. DenseNet has become one of the most common architectures in CNNs because it has a dense and interconnected structure. The DenseNet structure allows information to be preserved from disappearing or fading after passing through many layers. The idea lies in the basic principle of DenseNet, which is to ensure the flow of information by passing the output of each layer to all subsequent layers, as shown in Figure 2. This method assists in enhancing the quality of the features and makes the model easy to train [8,18]. DenseNet121 networks contain a total of 121 layers, including dense blocks with convolutional layers, batch normalization layers, and ReLU activation functions [19].

#### 3.2.2. VAE and β-VAE Models

**Figure 3:**
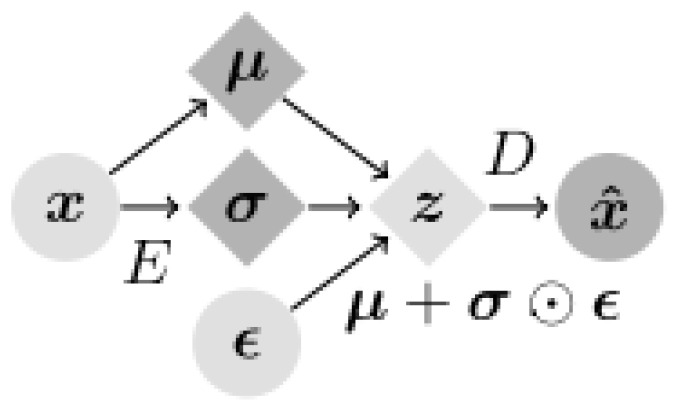
Variational autoencoder architecture.

Variational autoencoders (VAEs) are interesting in that they are one of the deepest generative models of unsupervised learning. A VAE is often used to generate low-dimensional latent space representations extracted from complex high-dimensional data. VAEs consist of encoders and decoders, where the encoder compresses the input data into a low-dimensional latent space, and the decoder reconstructs the original data from this latent space, as shown in Figure 3 [4,20,21]. The basic structure of the encoder includes four convolutional layers followed by two fully connected layers of 256 units. The latent distribution is a fully connected layer of 20 units that determines the mean and log standard deviation, in addition to using the ReLU activation feature and the Adam optimizer with a $5\times 10^{-4}$ learning rate [22]. In the Variational autoencoder (VAE) framework, two main types of losses are utilized: reconstruction error and Kullback–Leibler divergence (KLD).

The reconstruction loss evaluates the dissimilarity between the input data and the data reconstructed by the VAE model. In the first part of Equation (1), $q_{\theta }\left(z|x\right)$ represents the approximate posterior distribution of latent variables *z* given the input data *x*, parameterized by θ. On the other hand, $p_{\phi }\left(x|z\right)$ represents the likelihood distribution of generating *x* given a latent variable *z*. This aspect of the equation encapsulates the reconstruction process. The second part of Equation (1) measures the KLD between the approximate posterior distribution $q_{\theta }\left(z|x\right)$ and the prior distribution p(z) of the latent variable *z* [4].

(1) $$L_{\mathrm{VAE}}=-E_{q_{\theta }\left(z|x\right)}\left[\log p_{\phi }\left(x|z\right)\right]+\mathrm{KL}[q_{\theta }\left(z|x\right)||p\left(z\right)]$$

β-VAE is a modification of the variational autoencoder (VAE) framework made by adding a tunable parameter β. The purpose of the value of β is to assist in obtaining a disentangled latent representation. Larger β values often encourage more disentangled representations in the latent space [22]. Equation (2) introduces the incorporation of a factor β, where this factor (β≥0) is included to balance the reconstruction error and KLD losses [5,23,24].

(2) $$L_{\beta \text{-}\mathrm{VAE}}=L_{\mathrm{rec}}+\beta L_{\mathrm{KL}}$$

### 3.3. Proposed Method

**Figure 4:**
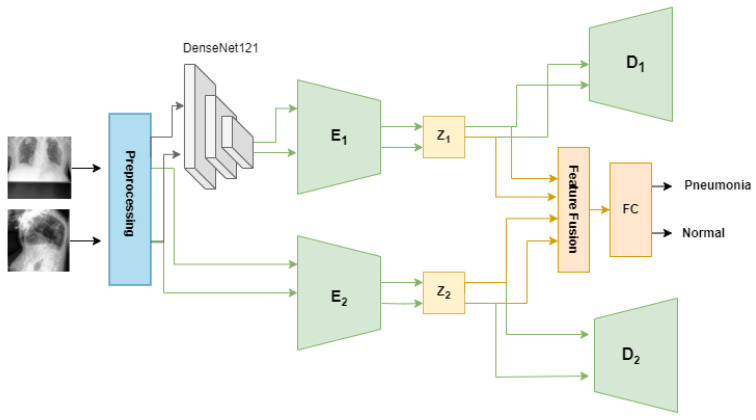
Structure of the proposed model. E1 and D1 are the encoder and decoder of the β-VAE. E2 and D2 are the encoder and decoder of the VAE.

The proposed method aims to classify X-rays into the presence or absence of pneumonia based on multi-view images. As shown in Figure 4, the proposed method has two steps, as follows:

(1) Preprocess the dataset.

In this step, the dataset is processed to ensure each patient has both frontal and lateral images, then resized and normalized to fit the proposed model.

(2) Train model.

This step provides a detailed explanation of the proposed MV-MFF model, highlighting the use of a VAE and a β-VAE to optimize the use of frontal and lateral images for pneumonia classification.

#### 3.3.1. Preprocess the Dataset

The dataset underwent preprocessing to prepare it for classification from multi-view. Originally, the dataset was structured to guarantee that every patient possessed both frontal and lateral images, eliminating those with only one image. In our organizational process, we employed folder numbers as distinctive identifiers for each patient, ensuring that each ID comprised both a frontal and a lateral image. Moreover, we adjusted the size to 224 × 224 [8,13]. To make them compatible with the backbones of β-VAE and VAE, we also added two channels to make the size 224 × 224 × 3. Then, the data are normalized by subtracting from the mean and then dividing by the standard deviation [13]. Due to the presence of uncertain labels in the dataset, various approaches exist to address them. Some studies leverage uncertain values by substituting them with positive or negative samples, while others opt to discard them entirely. In our experiment, we replaced uncertain labels with positive ones to maximize the utilization of available images. Additionally, any labels deemed as missing values were omitted from the dataset [12,17]. We obtained more than 6000 images from over 3000 patients, ensuring that each patient had both frontal and lateral views. The dataset included 4948 images of pneumonia and 1278 images of healthy cases.

#### 3.3.2. Train Model

There have been many generative models that seek to obtain useful and interpretable latent representations. The proposed MV-MFF model uses a VAE and a β-VAE to achieve better performance than that achieved individually. Combining multiple latent representations results in more comprehensive feature extraction, which contributes to the classification task effectively. This can help identify subtle patterns or associations that may not be captured by just one representation. This approach jointly exploits the use of the VAE and β-VAE to obtain latent representations capable of capturing the most important features that help in pneumonia classification, as shown in Figure 4. Initially, the model employs a pretrained DenseNet121 model as a feature extractor. Subsequently, these features, which are 1024-dimensional, are fed into the β-VAE to obtain disentangled latent representations. The encoder E1 of the β-VAE is trained to compute both the mean and the logarithm of the variance of the latent distribution within the latent space. These statistical measures are then utilized to derive a 32-dimensional latent space vector, denoted as z1, as shown in Equation (3). The decoder of the β-VAE reconstructs the original input based on the provided latent space vector z1.

(3) $$z_{i}=\mu_{i}+\sigma_{i}\epsilon$$

On the other hand, we used the VAE by passing the original images of size 224 × 224 × 3 to obtain complementary information that helps in the classification task. The VAE encoder E2 takes the original images and encodes them into the z2 latent space for both the frontal and lateral images. The encoder involves the utilization of three convolutional layers with 32-32-64 channels, 3-3-3 kernel size, and a stride of 2, each followed by batch normalization and LeakyReLU activation layers. Batch normalization helps to have a slight regularization effect, which can help prevent overfitting to some extent. The output from the convolutional layers is flattened and then passes through a sequence of fully connected layers. This includes three layers with 128 units each, two layers with 64 units each, and another two layers with 32 units each. Throughout this process, the LeakyReLU activation function is applied. After that, the mean and log variance of latent space are calculated, then a 32-dimension latent space vector z2 is produced. To fulfill the primary objective of pneumonia classification, all latent features present in both latent spaces z1 and z2 are leveraged. A fusion layer was added that combines the latent representations z1 and z2 using the concatenate function. The output of the fusion layer is fed to a fully connected layer with a softmax activation function to obtain a classification.

## 4. Experimental Setup

During the training phase, we meticulously fine-tuned various hyperparameters to ensure optimal performance for our specific models and dataset. For the VAE, we experimented with different batch sizes and settled on a batch size of 128. This choice was based on balancing computational efficiency with model convergence and stability. For the β-VAE variant, we opted for a slightly larger batch size of 256 to expedite training without sacrificing performance. Additionally, we employed the Adam optimizer and executed 50 epochs for both frontal and lateral image perspectives. This extensive training duration allowed the models to sufficiently learn the underlying representations of the data from different viewpoints. For the subsequent classification process, we adopted a learning rate of 0.001. By carefully tuning this parameter, we aimed to ensure stable and consistent training progress throughout the 200 epochs dedicated to classification. To evaluate the performance of our models, we partitioned the dataset into training and testing sets, allocating 80% of the data for training and reserving the remaining 20% for testing purposes. These specific parameters were selected after multiple experiments and evaluations to optimize performance.

## 5. Results

Expanding on the experimentation with various values of the parameter β sheds light on the nuanced trade-offs between disentanglement, reconstruction fidelity, and their impact on multi-view classification performance. β, as a key parameter in the β-VAE, plays a crucial role in balancing the reconstruction error and the KL divergence term in the VAE objective function. Table 2 compares how these factors affect classification accuracy and the AUC measure. Higher β values tend to produce more disentangled features. However, excessively high β values may lead to over-regularization, resulting in loss of detail and blurry images during reconstruction [5]. Conversely, lower β values prioritize reconstruction fidelity, preserving more image details but potentially sacrificing disentanglement. Based on the above, disentangled features may enhance interpretability and generalization, and blurry reconstructions may hinder practical applications requiring clear visual information. As a result, a β value of 0.001 was utilized in the remaining experiments, as it significantly contributed to the best results achieved.

**Figure 5:**
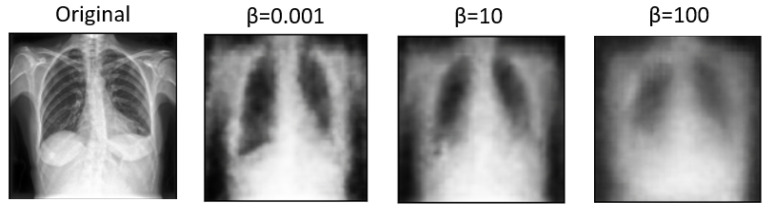
Images reconstructed using the β-VAE model without DenseNet121 with varying β values in frontal view.

**Figure 6:**
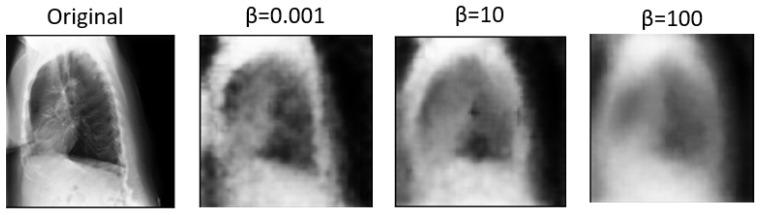
Images reconstructed using the β-VAE model without DenseNet121 with varying β values in lateral view.

**Figure 7:**
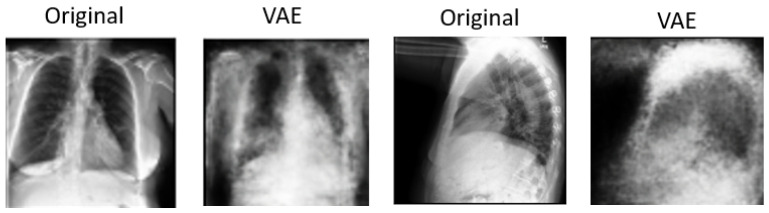
Images reconstructed using VAE model.

In Figure 5 and Figure 6, we showcase the reconstruction of original images using the decoder component of the β-VAE model, leveraging the latent space features z obtained from the encoder. These visualizations allow us to observe the effects of different β values on the quality and clarity of the reconstructed images. It becomes evident that as the β value increases, the reconstructed images tend to exhibit more blurriness. This phenomenon is a direct consequence of the trade-off between disentanglement and reconstruction fidelity, as discussed earlier. Despite the blurriness, crucial features and patterns from the original images remain discernible, indicating that the encoder successfully captured essential information during the encoding process. This underscores the robustness of the β-VAE framework in extracting meaningful latent representations from input data, even under the influence of regularization constraints imposed by higher β values [5,25]. Furthermore, Figure 7 provides a comprehensive view of the reconstruction process conducted by the decoder within the VAE model for both frontal and lateral images. By presenting these visualizations, we not only illustrate the impact of β values on reconstruction quality but also emphasize the efficacy of the VAE framework in capturing and preserving essential image features across different views. These insights contribute to a deeper understanding of the underlying mechanisms driving the performance of our proposed approach and underscore the importance of careful parameter selection in achieving optimal results.

**Figure 8:**
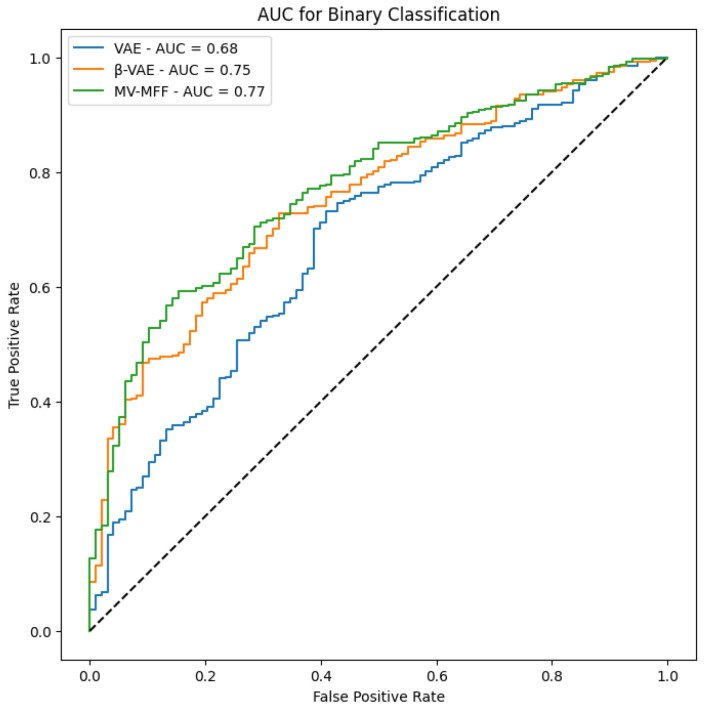
The AUC results of VAE, β-VAE, and MV-MFF on the CheXpert dataset.

In Figure 8, we present the AUC results obtained from evaluating the performance of each model. The AUC metric serves as a reliable measure of classification performance. Notably, the results demonstrate that the MV-MFF model consistently outperforms both the individual VAE and β-VAE models in terms of AUC. This superior performance can be attributed to several factors inherent to the MV-MFF model, including its ability to excel in effectively leveraging multi-view information by integrating features extracted from multiple perspectives. By incorporating insights from diverse viewpoints, the model gains a more comprehensive understanding of the underlying data distribution, leading to enhanced discriminative power and classification performance. In summary, the superior performance of the MV-MFF model, as evidenced by higher AUC scores, underscores its efficacy in leveraging multi-view information and integrating features from multiple models for more robust classification. These findings highlight the potential of multi-view learning approaches and feature fusion techniques in enhancing the discriminative power and performance of machine learning models across various applications and domains.

### Comparison with Similar Works

In conducting the performance comparison, we systematically evaluated our proposed MV-MFF model against prior studies that also utilized multi-view X-rays for pneumonia classification. The evaluation metric employed for this comparison was the AUC, which offers a robust measure of classification performance. In Table 3, we present a comprehensive comparison of our approach’s performance with that of previous studies in pneumonia classification. By leveraging both frontal and lateral X-ray views and utilizing latent representations with the CheXpert dataset, our MV-MFF model showcased notable improvements over existing methods. The key distinguishing factor of our approach lies in its utilization of latent representations, which enables the model to capture and encode complex patterns and relationships within the data more effectively. Unlike conventional approaches that may rely solely on handcrafted features or shallow representations, our model leverages the inherent expressiveness of latent representations learned through the training process. Moreover, the integration of multi-view information through our MV-MFF framework further augments the discriminative power of the model. The observed improvements in classification performance underscore the efficacy of our proposed approach in leveraging advanced machine learning techniques, such as multi-view learning and feature fusion, for pneumonia classification tasks. By surpassing the performance of previous methods, our approach demonstrates its potential to significantly contribute to the field of medical imaging and facilitate a more accurate and reliable diagnosis of pneumonia, ultimately benefiting patient care and decision making.

## 6. DeLong Test

The DeLong test is used to determine whether the differences between the AUCs of the models are statistically significant. When applying the DeLong test between each pair of models, a result with p<0.05 indicates a significant difference between the two models [26]. In this study, the DeLong test was applied to compare the statistical differences among the MV-MFF, VAE, and β-VAE models. The results obtained p>0.98, indicating that the models are not significantly different from each other.

Although the MV-MFF model showed a higher AUC of 0.775 compared to the VAE model of 0.68 and β-VAE model of 0.75, the DeLong test did not find significant differences between their AUCs. This suggests that despite a noticeable improvement in classification performance indicated by the higher AUC, the differences cannot be confirmed statistically within the dataset.

However, it is important to highlight that the higher AUC indicates better overall performance in distinguishing between classes, which can be crucial in practical settings and when compared with previous studies. Thus, even in the absence of statistically significant differences, the MV-MFF model’s superior AUC demonstrates its practical relevance and potential impact.

## 7. Ablation Study

### 7.1. Dependence on β-VAE

At this stage, our focus is solely on the β-VAE model independent of the VAE model. The chosen β-VAE model comprises two encoder units: one processing features extracted from DenseNet-121 with a dimensionality of 1024 and the other handling the original images. This model generates latent spaces, namely, z1 and z2, which are utilized in the classification process. The previous method yielded a classification outcome of 0.758 using the AUC metric. Based on the obtained results, it appears that the VAE model contributes positively to enhancing outcomes.

### 7.2. Impact of Feature Extraction Models

In our experimentation, we sought to explore the effectiveness of various convolutional architectures beyond DenseNet-121 in the feature extraction task. Table 4 presents a comprehensive comparison of the results obtained when replacing DenseNet-121 with alternative architectures, including DenseNet-169, DenseNet-201, InceptionV3, VGG19, ResNet50, and ResNet101, for the same purpose. The classifier findings derived from these experiments offer valuable insights into the comparative performance of each architecture. It becomes evident that while all architectures contribute to the classification task, there are notable variations in their effectiveness in terms of AUC score and accuracy. Specifically, DenseNet-121 emerges as the top-performing architecture, outperforming other models in terms of AUC score. This indicates that DenseNet-121 effectively captures and represents discriminative features from the input images, leading to superior classification performance. The dense connectivity pattern in DenseNet architectures likely contributes to its effectiveness in extracting relevant features and facilitating more robust classification. Additionally, both DenseNet-121 and ResNet-50 demonstrate superior accuracy compared to other feature extraction models. In summary, these findings underscore the critical significance of choosing the right feature extraction model to enhance classification performance effectively.

### 7.3. Compared with Single View

To prove the significance of multi-view approaches in enhancing classification quality and diagnostic reliability, we conducted experiments using only a single view. Table 5 illustrates a comparison of utilizing either the frontal view alone, the lateral view separately, or a combination of both views based on the AUC measure. MV-MFF achieves the highest AUC score of 0.775, indicating a substantial enhancement in classification quality compared to utilizing either view in isolation. This highlights the synergistic benefits of integrating complementary information from multiple perspectives, resulting in a more comprehensive model.

## 8. Discussion

**Figure 9:**
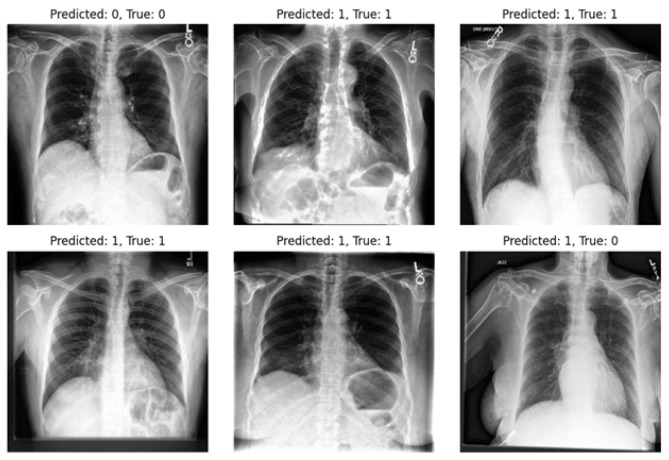
Utilizing a classifier to predict frontal images from the test set.

**Figure 10:**
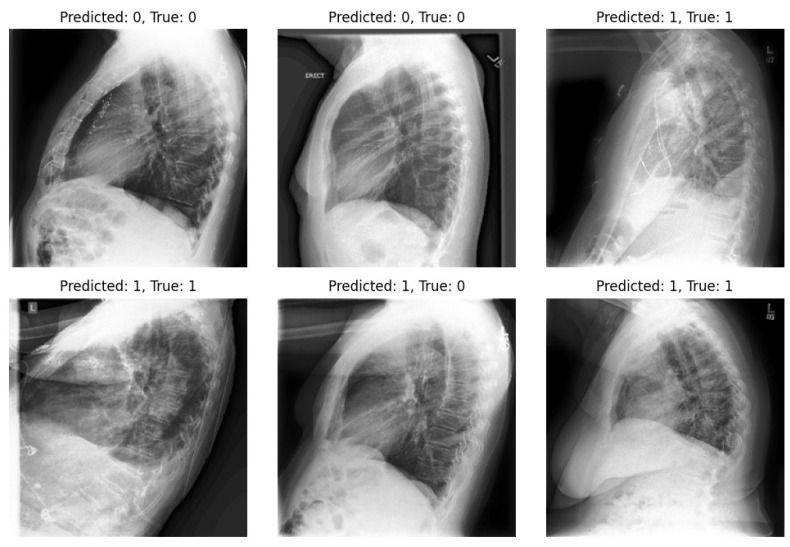
Utilizing a classifier to predict lateral images from the test set.

Our study underscores the pivotal role of multi-view X-rays in augmenting the reliability of disease diagnosis, particularly in the case of pneumonia. Our experiments validate the significance of incorporating lateral-view X-rays alongside frontal ones for more comprehensive assessments, thereby enhancing the overall reliability of the diagnostic process and facilitating more confident clinical decisions. By integrating latent representations derived from both the VAE and β-VAE models, our approach significantly enhances diagnostic outcomes in pneumonia classification tasks. On the other hand, we highlight one of the limitations of our experiments: the imbalance within our dataset. Notably, in the experiments, the number of positive samples was twice that of negative samples. This imbalance stemmed from the scarcity of images devoid of the disease and offering diverse viewpoints within the dataset. The classifier’s performance is illustrated in Figure 9 and Figure 10, revealing instances where the model erroneously classified negative images as positive when evaluating the test set. Despite these challenges, our methodology shows promising performance and yields satisfactory results compared to previous studies.

The experimental platform utilized in this article was the Colab Pro notebook environment. The system specifications included 50 GB of RAM, a GPU with 15 GB, and additional disk space. Given these resource limitations, it was not feasible to scale up the computational workload for extended periods. The Python code was executed using version 3.10.12, and the TensorFlow framework was employed for implementation. Several libraries were utilized, including Pandas, NumPy, Matplotlib, Keras, and Scikit-learn.

## 9. Conclusions

We presented a model capable of distinguishing the presence or absence of pneumonia using both frontal and lateral views, aiming to achieve a more reliable diagnosis. Our results demonstrate that incorporating lateral views alongside frontal ones improves classification quality compared to using either view separately. The proposed MV-MFF model leverages latent representations extracted from encoders in both a VAE and a β-VAE, effectively combining them for classification. This approach involves extracting useful and meaningful representations from the input data. In this study, we investigated the effect of varying the β value on our results. Unlike with higher values, we found that a β value of 0.001 yielded better results in the classification process. The β-VAE model achieved 80.8% accuracy and an AUC of 0.75, while the VAE achieved an AUC of 0.68. Our experiments were conducted using the CheXpert dataset, which contains multi-view images. The MV-MFF model achieved an AUC of 0.74 for the frontal view and 0.71 for the lateral view. Combining latent representations from frontal and lateral images led to an accuracy of 80.4% and an AUC of 0.775. These results underscore the valuable additional information provided by lateral images, contributing to a more comprehensive understanding of the disease. To statistically compare the performance of our models, we applied the DeLong test, which showed no significant difference between the AUCs of the models.

In future work, we plan to explore other lung and heart diseases included in the dataset. Additionally, we aim to address the limitations of our current approach by expanding the data and implementing further improvements.

## Figures and Tables

**Table 1:** Literature survey.

References	Findings	Method	Dataset	View	AUC
Janizek	Generalization of the	Adversarial	CheXpert+	Frontal	0.747
et al. [11]	data outside the	approach	MIMIC-CXR	(pneumonia)	0.739
	training set for pneumonia				
	classification				
Zhu et al. [10]	Multi-view	DenseNet121+	MIMIC-CXR	Frontal	0.693
	classification	back-projection		Lateral	0.669
		transfer		Multi-view	0.715
				(pneumonia)	
Rubin et al. [9]	Multi-view	DenseNet121	MIMIC-CXR	PA–lateral	0.625
	classification			AP–lateral	0.593
				(pneumonia)	
Ma et al. [12]	Multi-view	Cross-attention	CheXpert	Multi-view	0.666
	classification	networks		(pneumonia)	
Peng et al. [8]	Multi-view	DenseNet121+ InceptionV3	MIMIC-CXR	Multi-view	0.71
	classification	+Xception		(pneumonia)	
Tulder et al. [13]	Multi-view	ResNet-18+	CheXpert	Frontal	0.75
	classification	cross-view		Lateral	0.74
		transformer		Late joining	0.76
				Cross-view	0.75
				(pneumonia)	
Kim [14]	Medical image	ConvNeXt +	MIMIC-CXR	Multi-view	0.85
	classification	transformer		(26 classes)	
Yuan et al. [15]	Generating radiology	LSTM +	CheXpert +	Multi-view	0.76
	reports	Resnet-152	Chest X-ray	(14 classes)	
Yang et al. [16]	Cardiovascular disease	Bidirectional image	National Lung	Multi-view	0.8243
	prediction (CVD)	Mamba (BI-Mamba)	Screening Trail	(CVD)	

**Table 2:** Performance evaluation of β-VAE using various β values.

β	Accuracy %	AUC
0.001	80.8	0.756
0.1	80.4	0.748
1	78.8	0.67
2	80	0.65
5	80.4	0.57
10	80.2	0.526
100	80.4	0.502

**Table 3:** A comparison of the proposed method of pneumonia classification with similar works.

Model	AUC
Cross-attention networks (CAN) [12]	0.666
Cross-view transformers [13]	0.754
Late join [13]	0.766
MV-MFF (ours)	0.775

**Table 4:** Comparison of classifier performance utilizing various feature extractor models.

Model	Accuracy %		AUC	
	β **-VAE**	**MV-MFF (Ours)**	β **-VAE**	**MV-MFF (Ours)**
DenseNet121	80.8	80.4	0.75	0.775
DenseNet169	79.6	79.8	0.739	0.746
DenseNet201	79.4	79.6	0.703	0.726
ResNet50	80.4	81.6	0.679	0.703
ResNet101	78.8	79.4	0.649	0.697
VGG19	78.4	79.6	0.708	0.731
InceptionV3	79.4	80,6	0.70	0.708

**Table 5:** Comparison of the experimental outcomes attained by utilizing a sole viewpoint, assessed through the AUC measure.

Model	Frontal View	Lateral View	Multi-View
VAE	0.64	0.63	0.68
β-VAE	0.71	0.70	0.75
MV-MFF (ours)	0.74	0.71	0.775

## Data Availability

The CheXpert dataset is available online upon request from the official Stanford ML Group website via the link https://stanfordmlgroup.github.io/competitions/chexpert (accessed on 7 December 2022).

## Abbreviations

List of nomenclature and abbreviations:

MV-MFF	Multi-view multi-feature fusion model
VAE	Variational autoencoder
β-VAE	β-bariational Autoencoder
CNN	Convolutional neural network
AUC	Area under the curve
KLD	Kullback–Leibler divergence
